# Retraction Note: Regenerative potentials of bone marrow mesenchymal stem cells derived exosomes or its combination with zinc in recovery of degenerated circumvallate papilla following surgical bilateral transection of glossopharyngeal nerve in rats

**DOI:** 10.1186/s12903-025-07383-3

**Published:** 2025-12-19

**Authors:** Eman Mohamed Salem, Hamdy Rizk, Yara S. Abouelela, Abdelbary Prince, Adel Fathy Tohamy, Nawal A. Lasheen, Bassant A. Ezzat, Sana Mostafa

**Affiliations:** 1 Oral Biology Department, College of Oral and Dental Surgery, Misr University for Science and Technology, Giza, 12568 Egypt; 2 Oral Biology Department, Faculty of Dentistry, Cairo University, Cairo, 115533 Egypt; 3 Anatomy and Embryology Department, Faculty of Veterinary Medicine, Cairo University, Giza, 12211 Egypt; 4 Department of Biochemistry and Molecular Biology, Faculty of Veterinary Medicine, Cairo University, Giza, 12211 Egypt; 5 Department of Toxicology and Forensic Medicine, Faculty of Veterinary Medicine, Cairo University, Giza, 12211 Egypt

**Retraction Note: BMC Oral Health 24, 1320 (2024)**

**https://doi.org/10.1186/s12903-024-05050-7**

The Editor has retracted this article. A number of image integrity concerns were raised after publication. In Fig. 1A, there appear to be several areas of overlap within the micrograph. In Fig. 1C, two graphs appear to overlap and are presented as different experimental conditions. The authors have been unable to provide a sufficient response to concerns. The Editor has lost confidence in the results and conclusions of this article.

Authors Hamdy Rizk, Bassant A. Ezzat, Sana Mostafa, Eman Mohamed Salem and Nawal Abdelkhalek Lasheen disagree with this retraction. Authors Yara S. Abouelela, Abdelbary Prince and Adel Fathy Tohamy did not respond to correspondence regarding this retraction.

